# Distribution and prevalence of Sarcina troglodytae in chimpanzees and the environment throughout Africa

**DOI:** 10.1099/jmm.0.002044

**Published:** 2025-07-18

**Authors:** Emily Dunay, Ismail Hirji, Leah A. Owens, Konkofa Marah, Naomi Anderson, Maria Ruiz, Rebeca Atencia, Joshua Rukundo, Alexandra G. Rosati, Megan F. Cole, Melissa Emery Thompson, Jacob D. Negrey, Samuel Angedakin, Johanna R. Elfenbein, Tony L. Goldberg

**Affiliations:** 1 Department of Pathobiological Sciences, School of Veterinary Medicine, University of Wisconsin-Madison, Madison, WI, USA; 2 Tacugama Chimpanzee Sanctuary, Freetown, Sierra Leone; 3 Jane Goodall Institute Congo, Pointe-Noire, Republic of Congo; 4 Ngamba Island Chimpanzee Sanctuary/Chimpanzee Trust, Entebbe, Uganda; 5 Department of Psychology, University of Michigan, Ann Arbor, MI, USA; 6 Department of Anthropology, University of Michigan, Ann Arbor, MI, USA; 7 Department of Anthropology, University of New Mexico, Albuquerque, NM, USA; 8 School of Anthropology, University of Arizona, Tucson, AZ, USA; 9 Department of Environmental Management, Makerere University, Kampala, Uganda

**Keywords:** bacteria, chimpanzee, epidemiology, sanctuary, *Sarcina*, Sierra Leone

## Abstract

**Introduction.** Since 2005, the leading cause of death for western chimpanzees (*Pan troglodytes verus*) at Tacugama Chimpanzee Sanctuary (TCS) in Sierra Leone has been epizootic neurologic and gastroenteric syndrome (ENGS), associated with the bacterium *Sarcina troglodytae* (family *Clostridiaceae*).

**Gap Statement.** The prevalence of *S. troglodytae* at TCS in clinically normal chimpanzees and the environment remains unknown, as does its distribution in other captive and wild chimpanzee populations and their environments across Africa.

**Aim.** The aim of this study was to determine the distribution and prevalence of *Sarcina* bacteria in sanctuary and wild chimpanzee populations across Africa and to identify demographic and ecological risk factors for *S. troglodytae* in chimpanzees and the environment.

**Methodology.** We conducted a prospective, multi-season epidemiological investigation of *S. troglodytae* in chimpanzees and the environment at TCS and a parallel study at a sanctuary in the Republic of Congo. We also describe the results of surveys of chimpanzees at a sanctuary in Uganda and wild chimpanzee populations in Sierra Leone and Uganda for *S. troglodytae*. In total, we tested 637 chimpanzee and environmental samples using a species-specific PCR for *S. troglodytae* and a pan-*Sarcina* PCR.

**Results.** *S. troglodytae* was more prevalent in chimpanzees at TCS (*n*=60) during the dry season (96.7%) than during the rainy season (55.2%). Soil was the most common environmental source of the bacterium (54% dry season vs. 4.8% rainy season). Notably, we did not detect *S. troglodytae* in faecal samples from sanctuary chimpanzees in the Republic of Congo (*n*=79) or in wild chimpanzees in Sierra Leone (*n*=18). We did detect the bacterium in East African chimpanzees (*n*=84) but at low prevalence (2.6%–10.9%). In contrast, we found the genus *Sarcina* to be ubiquitous in all chimpanzee populations with a higher prevalence in sanctuary chimpanzees (93.1%–100%) than in wild chimpanzees (66.7%–68.4%).

**Conclusion.** *S. troglodytae* is markedly more prevalent at TCS, the only location affected by ENGS, than at any other location tested, and soil is a likely reservoir of *S. troglodytae*. These findings strengthen the association between * S. troglodytae* and ENGS and have implications for sanctuary management and conservation of western chimpanzees.

## Data Summary

Sequence data generated in this study have been deposited in GenBank under accessions PQ092974–PQ092985. Supplementary figures and tables are available with the online version of this article.

## Introduction

Infectious disease is a threat to endangered great ape populations across Africa and Asia [1,4]. Significant attention has been directed towards viral threats to great apes including polio-like viruses (genus *Enterovirus*) [5,6], poxviruses [7,8], simian immunodeficiency viruses (SIVs) [9,12], ebolaviruses [13,15] and common cold viruses, namely, rhinovirus C [16], metapneumovirus [17,21], respiratory syncytial virus [18,22,25], respirovirus/parainfluenza virus 3 [19] and coronavirus OC43 [26]. These concerns have been heightened by the emergence of SARS-CoV-2 [27,28], which has been transmitted to great apes in captivity [29,31]. However, bacterial diseases also impact great ape populations [32]. For example, anthrax has been documented in wild chimpanzees (*Pan troglodytes*) and gorillas (*Gorilla gorilla*) in Côte d’Ivoire and Cameroon [33,35]. During viral respiratory outbreaks, co-infection with bacterial pathobionts, most commonly *Streptococcus pneumoniae*, can contribute to mortality [22,24, 36]. *Treponema pallidum* subspecies *pertenue* and *Mycobacterium leprae*, the causative agents of yaws and leprosy, respectively, have been detected in diseased, wild western chimpanzees (*P. t. verus*) in West Africa, which are critically endangered [37,39].

The genus *Sarcina*, which contains two recognized species (*Sarcina ventriculi* and *Sarcina maxima*), has been described in the gut microbiota of many clinically normal animals including humans [40,41] and non-human primates (NHPs) [42] and is ubiquitous in the environment [43]. However, sarcinae have also been increasingly associated with gastrointestinal illness in humans in which the type species, *S. ventriculi*, is thought to be an opportunistic pathogen associated with delayed gastric emptying [44,45]. Recently, in a population of rhesus macaques (*Macaca mulatta*) in the USA, *Sarcina* in non-alimentary tissues was associated with gastric distension, gas accumulation and unexpected death, in a clinical outbreak evocative of ENGS, but organism identity could only be confirmed at the genus level [46]. Similarly, *S. ventriculi* was identified in the stomach and duodenum of a laboratory-housed cynomolgus macaque (*Macaca fascicularis*) in Korea that died suddenly from acute gastric dilation and rupture (47). Reports of *Sarcina*-associated disease in chimpanzees are limited to our previous study [44] discussed below; however, in a longitudinal gut microbiome study of six wild, habituated eastern chimpanzees (*P. t. schweinfurthii*) at Gombe National Park in Tanzania, an increase in the relative abundance of the genus *Sarcina* was observed after natural infection with SIV [48]. Interestingly, *Sarcina* was not detected at a high abundance in 35 SIV-negative individuals from this population [49].

Previously, we identified a novel bacterium, *Sarcina troglodytae* (family *Clostridiaceae*), in a population of wild-born, sanctuary-housed western chimpanzees at Tacugama Chimpanzee Sanctuary (TCS) in Sierra Leone [44]. Using a case-control study design, we found an epidemiologic association between *S. troglodytae* and epizootic neurologic and gastroenteric syndrome (ENGS), which has affected at least 56 chimpanzees at TCS since 2005 resulting in death in all cases, the majority of which have occurred during the dry season in the month of March. To our knowledge, ENGS has not been documented in captive or wild chimpanzee populations outside of TCS. This observation, the lack of known human cases of similar disease near TCS and the propensity for clostridia to persist in the environment [50] suggest that characteristics of the physical environment inhabited by chimpanzees at TCS might contribute to the persistence of *S. troglodytae* and ENGS. The goals of this study were therefore to identify potential environmental sources of *S. troglodytae* at TCS and to determine whether *S. troglodytae* is present in captive and wild chimpanzee populations other than those at TCS.

Here, we describe a study of the distribution of *S. troglodytae* in chimpanzee populations across Africa, its prevalence in chimpanzees and the environment and risk factors for infection in chimpanzees. At TCS, we conducted an ecological and epidemiological investigation of the bacterium in chimpanzees and the environment during the dry (December–April) and rainy (May–November) seasons. Our goal was to describe the prevalence of the bacterium and identify demographic and ecological risk factors for the presence of *S. troglodytae*. For comparison, we conducted a similar study at Tchimpounga Chimpanzee Rehabilitation Center (TCRC), a sanctuary located in the Republic of Congo and within the range of central chimpanzees (*P. t. troglodytes*). For comparison of a wild chimpanzee population to the captive chimpanzees at TCS, we tested unhabituated, western chimpanzees in Loma Mountains National Park (LMNP) in Sierra Leone. In addition, for a more distant geographical comparison, we tested archived faecal samples from eastern chimpanzees at Ngamba Island Chimpanzee Sanctuary (NICS) and two communities in Kibale National Park (KNP) in Uganda.

Our findings shed new light on the distribution and prevalence of *S. troglodytae*, in particular, and of the genus *Sarcina* in general*,* across captive and wild chimpanzee populations within the natural chimpanzee range, including the three most populous subspecies (i.e. western, central and eastern chimpanzees). We also identify demographic and ecological factors associated with *S. troglodytae* presence in chimpanzees and the environment. Ultimately, our findings inform ongoing ENGS mitigation efforts at TCS and carry broader implications for potential health risks associated with efforts by sanctuaries to aid the conservation of wild populations by conducting translocations or reintroductions.

## Methods

### Study sites

**Fig. 1.**
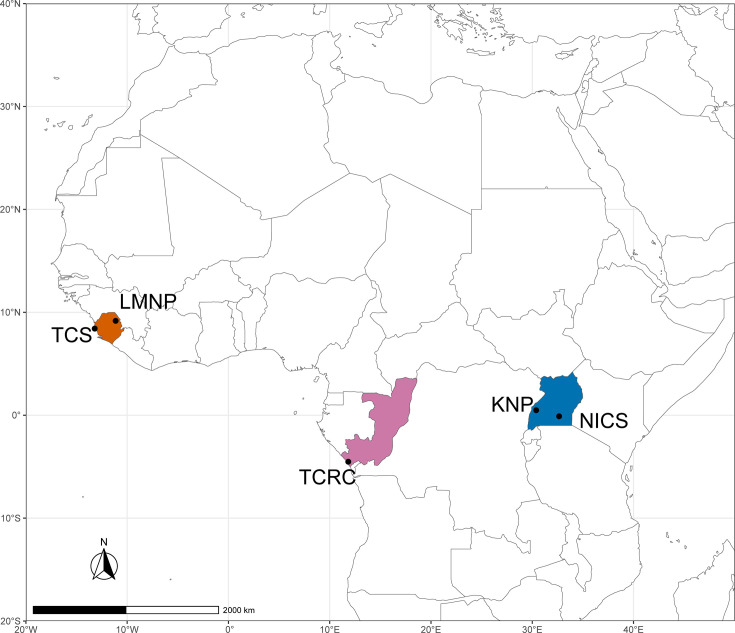
Map of study sites. Study sites included TCS and LMNP in Sierra Leone (orange), TCRC in the Republic of Congo (pink) and KNP and NICS in Uganda (blue). The map was created using packages ‘ggplot2’ and ‘rnaturalearth’ using R v. 4.2.0 in RStudio [56,57].

First, we conducted a multi-season ecological and epidemiological study of chimpanzees and the environment at TCS, located in Western Area Peninsula National Park, Sierra Leone, in West Africa (Fig. 1). TCS cares for ~118 chimpanzees who, after quarantine and rehabilitation, are integrated into one of eight social groups, each with a separate enclosure (either non-forested or forested) and dormitories where they spend the night. For comparison, we conducted a similar field study at TCRC, another Pan African Sanctuary Alliance member, located within the Tchimpounga Nature Reserve, north of Pointe-Noire, Republic of Congo, in Central Africa. TCRC cares for ~150 chimpanzees, including a main sanctuary site with several enclosures (non-forested and forested) and three forested islands in the Kouilou River on which chimpanzees roam freely during the day but return to dormitories at night. Additionally, we tested chimpanzee faecal samples from a third sanctuary, NICS, located on Ngamba Island in Lake Victoria, Uganda, in East Africa, which cares for ~52 chimpanzees that semi-free range in a 100-acre forested enclosure and return to dormitories at night. At all sanctuary sites, chimpanzees are fed seasonally available fruits, vegetables and other foods several times per day, and they also forage within the forested enclosures.

To investigate the occurrence of *S. troglodytae* outside of sanctuaries, we also studied two wild chimpanzee populations. LMNP in Sierra Leone’s Northern Province is home to an estimated 1,002 unhabituated, wild western chimpanzees [51]. This population provides an informative comparison to TCS, because both are in the range of the western subspecies of chimpanzee, *P. t. verus*, and are only ~250 km apart. At LMNP, we collected chimpanzee faecal and environmental samples for direct comparison to TCS. For a more geographically distant comparison, we tested archived chimpanzee faecal samples from KNP in western Uganda, home to multiple habituated eastern chimpanzee communities including the two studied here, Kanyawara and Ngogo. At the time of the study, these communities contained ~55 and 204 chimpanzees, respectively [52].

### Sample populations and sample collection

At TCS, we collected faeces from 60 wild-born chimpanzees (31 females and 29 males; estimated ages 1 to 40 years) between March and April of 2023 (dry season), and again, from 58 of the same individuals, correspondingly, between October and November of 2023 (rainy season; two individuals were unable to be sampled in the rainy season). Individuals from all chimpanzee groups at TCS (including quarantine) were included to ensure a representative sample population. Researchers or sanctuary staff collected faecal samples from chimpanzees who had been isolated in a den overnight or directly observed to defecate, such that the individual’s identity was known. We used a clean wooden tongue depressor to transfer faeces into a sterile sample collection tube, making sure to sample from the centre of the bolus to avoid environmental contamination. In addition, we collected soil samples (*n*=63), water samples (*n*=24) and food samples (*n*=20) between March and April of 2023 and again, from the same sites for soil and water, between October and December of 2023.

We collected soil samples directly from the top layer of soil with a clean wooden tongue depressor and placed them into sterile collection tubes. We sampled each enclosure at TCS, non-forested (*n*=4) and forested (*n*=5), using a randomized grid sampling design generated by the mobile application Soil Sampler (Farmis), adjusting the density of the sampling points such that seven soil samples were collected from each enclosure. We collected water samples from inside each enclosure from either standing water sources or taps used to provide water to chimpanzees and throughout the sanctuary grounds (tap, tank, reservoir, handwash station and stream). We used a 50 ml sterile Luer Lock syringe (Becton Dickinson, Franklin Lakes, NJ, USA) affixed with a 25 mm Swinnex filter holder (MilliporeSigma, Burlington, MA, USA) into which was placed a 1.2 um polycarbonate Isopore membrane filter (MilliporeSigma). Water was pushed through the membrane filter until a total volume of 200 ml was reached or until maximum resistance was met.

We sampled a variety of chimpanzee food items (i.e. fruits and vegetables, before and after washing, and vegetation in enclosures known to be eaten by chimpanzees) using sterile polyester-tipped swabs (SteriPack, Lakeland, FL, USA). We first wetted the swab with sterile PCR-grade water (Ambion, Austin, TX, USA) and then ran the swab for 10–15 s over the surface of a single food item in the case of fruits and vegetables or over five leaves from a single stem in the case of vegetation. We stored fresh samples at room temperature and processed them within 6 h of collection. When samples could not be processed within 6 h, we stored them in RNAlater nucleic acid preservation buffer (Thermo Fisher Scientific, Waltham, MA, USA) at a 1 : 1 ratio for up to a week prior to DNA extraction or kept them at −80 °C if longer storage was necessary (Table S1, available in the online Supplementary Material).

At TCRC, sanctuary staff collected faecal samples from 79 individuals (36 females and 43 males; estimated ages <1–37 years old), either directly from the rectum using a gloved hand during routine health examinations under anaesthesia or opportunistically as described above for TCS. Additionally, sanctuary staff collected faecal samples from 17 Old World monkeys housed at the sanctuary including four vervets (*Chlorocebus* sp.), nine mandrills (*Mandrillus sphinx*), one collared mangabey (*Cercocebus torquatus*), one greater spot-nosed guenon (*Cercopithecus nictitans*), one moustached guenon (*Cercopithecus cephus*) and one talapoin (*Miopithecus* sp.) opportunistically. We generated soil sampling points as randomized grids for the three islands (*n*=66) and four of the enclosures at the main sanctuary (*n*=28; seven samples per enclosure) using QGIS 3.28.7, and sanctuary staff collected soil samples at those points. We collected water samples (*n*=9) and food samples (*n*=18) from the islands and main sanctuary, as described for TCS. Fresh samples were frozen at −20 °C on the day of collection until processing. Samples collected in RNAlater at a 1 : 1 ratio were stored at room temperature for up to 1 week or at −20 °C prior to processing (Table S1). All TCRC samples were collected between February and March 2024 (rainy season).

For NICS, we tested archived faecal samples from 46 wild-born chimpanzees (27 females and 19 males, estimated ages 8–34 years old) previously collected during routine health checks during July and August of 2017 (dry season), as described for TCRC. Samples were frozen in liquid nitrogen, kept frozen during shipment to the USA and then stored at −80 °C until processing. For KNP, we tested archived faecal samples from 38 habituated, wild eastern chimpanzees (Kanyawara females=8 and males=10, ages 9–51 years old; Ngogo females=10 and males=10; ages 13–67 years old) collected between June and December 2017 (dry and rainy seasons), as previously described [52]. Faecal samples were collected immediately after an individual was observed to defecate and stored in RNAlater at a 1 : 1 ratio. Samples were stored at −20 °C until transport on ice to the USA and then stored at −20 °C until processing in Madison, WI. At LMNP, park personnel collected 18 chimpanzee faecal samples in November of 2023. As the chimpanzees at LMNP are unhabituated, individual identities and demographic information are unknown. Two of the LMNP faecal samples (Table S1) were dried with silica and stored in ethanol at room temperature for a maximum of 3 weeks until processing, at which time samples were centrifuged at 8,000 r.p.m. for 3 min and ethanol was removed and allowed to evaporate and washed with 1 ml of sterile PBS prior to extraction [53]. The remaining 16 faecal samples from LMNP were collected in RNAlater at a 1 : 1 ratio and stored at room temperature for up to 1 week prior to processing. Concurrently, park personnel collected 19 soil samples from ~5 m away from a faecal collection site. All LMNP soil samples were collected in RNAlater at a 1 : 1 ratio and stored at room temperature for up to 1 week prior to processing.

### DNA extraction and PCR testing

We extracted DNA from all samples using the PowerLyzer PowerSoil DNeasy Kit (Qiagen, Hilden, Germany). At TCS, TCRC and LMNP, we processed samples on site using portable laboratory equipment. We mechanically lysed samples in the field using the Portalyzer at speed 2 for 15 min [54]. For NICS and KNP samples, we mechanically lysed samples using a Mini-Beadbeater (BioSpec Products, Bartlesville, OK, USA) for 30 s. We then conducted subsequent DNA extraction steps according to the manufacturer’s protocols.

To test samples for *S. troglodytae*, we designed specific PCR primers to amplify a 243 bp portion of a capsular biosynthesis gene of the bacterium (GenBank acc. no. CP051754; locus_tag=HH195_01965): Strog_SCBP_F (Tm=57 °C) and Strog_SCBP_R (Tm=55.3 °C) (IDT, Coralville, IA, USA). This gene is present in *S. troglodytae* but not in related *Sarcina* species [44], and the amplicon sequence is not found in other described organisms. Additionally, we tested all samples with pan-*Sarcina* primers, as previously described [44]. The pan-*Sarcina* primers target the V2–V3 region of the 16S rRNA gene of all three members of the genus *Sarcina*: TacuSarc_Diag_F (Tm=52.8 °C) and TacuSarc_Diag_R (Tm=53 °C) (IDT) (Fig. S1). We performed PCRs in 25 µl volumes containing 0.2 µM of each primer, 12.5 µl of 2× HotStar Master Mix (Qiagen), 9.5 µl of PCR-grade water and 2 µl of template DNA under the cycling conditions described in Table 1. The lower limit of detection for the *S. troglodytae* primers designed in this study was determined using a gBlocks gene fragment (IDT) of the target amplicon (243 bp).

**Table 1.** Primers used in this study

Primer set	*S. troglodytae*	Pan-*Sarcina*
**Forward primer**	Strog_SCBP_F5′-ATGCACTAGGGGCAAATGGAA-3′	TacuSarc_Diag_F5′-TGAAAGGCATCTTTTAACAATCAAAG-3′
**Reverse primer**	Strog_SCBP_R5′-TTGGCGAAACTGGTGTTTGA-3′	TacuSarc_Diag_R5′-TACCGTCATTATCGTCCCTAAA-3′
**PCR conditions**	95 °C for 15 min; 38 cycles of 94 °C for 45 s, 58 °C for 45 s and 72 °C for 60 s; and 72 °C for 10 min	95 °C for 15 min; 38 cycles of 94 °C for 45 s, 48 °C for 45 s and 72 °C for 60 s; and 72 °C for 10 min
**Gene target**	Capsular biosynthesis	16S rRNA
**Amplicon length (bp**)	243	289
**Reference**	This study	[44]

We performed PCR of TCS, TCRC and LMNP samples in the field on a mini16 (miniPCR bio, Cambridge, MA, USA) or Bento Lab Pro (Bento Bioworks, London, UK) thermal cycler. We visualized PCR products on 2% agarose gels with GelGreen nucleic acid stain (Biotium, Fremont, CA, USA) and 1 kb Plus DNA length standards (New England Biolabs, Ipswich, MA), using a blue light imager (Bento Bioworks). For samples from KNP and NICS, we performed PCR on a T100 thermal cycler (Bio-Rad, Hercules, CA, USA) and visualized PCR products on 2% agarose gels stained with ethidium bromide and 1 kb Plus DNA length standards (New England Biolabs) using a UV light GelDoc XR imager (Bio-Rad). We Sanger-sequenced amplicons on ABI 3730xl DNA Analyzers (Applied Biosystems, Foster City, CA, USA) at the University of Wisconsin-Madison Biotechnology Center as previously described [44].

### Statistical analyses

We calculated the prevalence of *S. troglodytae* and the genus *Sarcina* as the proportion of positive samples, with modified Wald 95% CIs [55]. We calculated odds ratios with 95% CIs and assessed significance using Fisher’s exact tests (function ‘fisher.test’ from package ‘stats’ in R, two-tailed) to describe associations between sex (male or female) and *S. troglodytae* or genus *Sarcina* presence at each study site. We used binomial generalized linear models (GLMs; function ‘glm’ from package ‘stats’ in R) to examine the effects of demographic (age and sex) and ecological (enclosure type, season and study site) predictor variables on *S. troglodytae* and genus *Sarcina* presence or absence in chimpanzee faecal samples. We also used binomial GLMs to assess the effect of ecological predictor variables (sample type, season and study site) on *S. troglodytae* and *Sarcina* presence or absence in environmental samples. We reported odds ratios with 95% CIs from logistic regression results calculated as the exponent of the beta coefficient (*β*) or, when applicable, the inverse [i.e. 1/(exp(*β*))]. For all statistical tests, we set the significance level (alpha) to 0.05. Data were analysed using R v. 4.2.0 implemented in RStudio v. 2023.06.1 [56,57].

## Results

### TCS

**Fig. 2.**
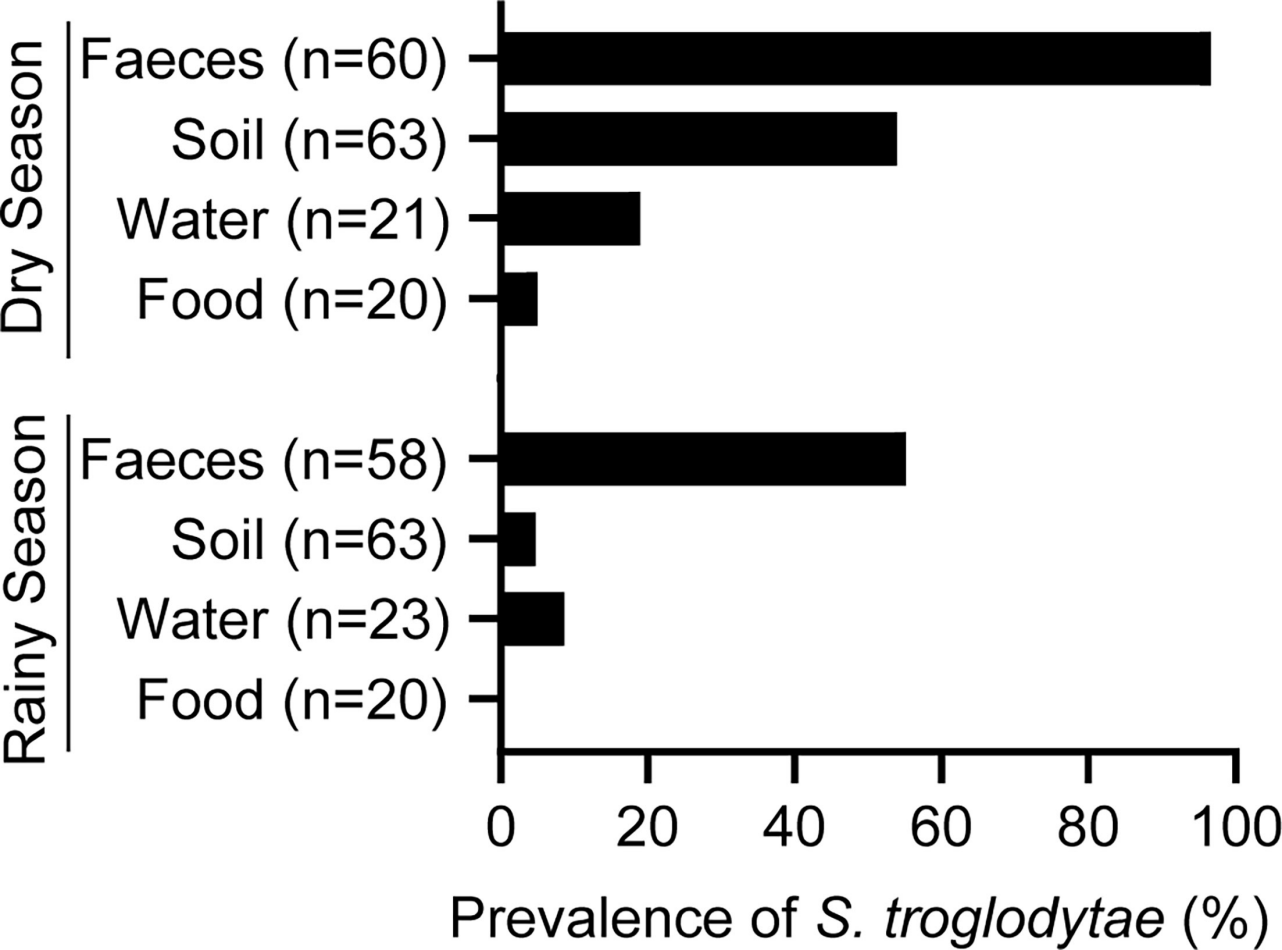
Prevalence of *S. troglodytae* in faeces and the environment at TCS during the dry and rainy seasons of 2023.

In total, we tested 118 chimpanzee faecal samples, 126 soil samples, 44 water samples and 40 food samples from TCS. The overall prevalence of *S. troglodytae* was 76.3% (95% CI: 67.8, 83.09) in chimpanzee faeces, 29.4% (95% CI: 22.1, 37.86) in soil, 13.6% (95% CI: 6.02, 27.09) in water and 2.5% (95% CI: <0.01, 20.19) in food. We observed seasonal differences in *S. troglodytae* prevalence in chimpanzees and the environment (Fig. 2, Tables S2 and S3).

At least one individual from quarantine and at least one individual in each of the eight chimpanzee groups at TCS were positive for *S. troglodytae* during the dry season. At least one individual in each of the eight chimpanzee groups at TCS was also positive for *S. troglodytae* during the rainy season; however, neither of the two individuals in quarantine during the rainy season was positive. There was no association between sex and *S. troglodytae* detection at TCS (Table S2).

**Fig. 3.**
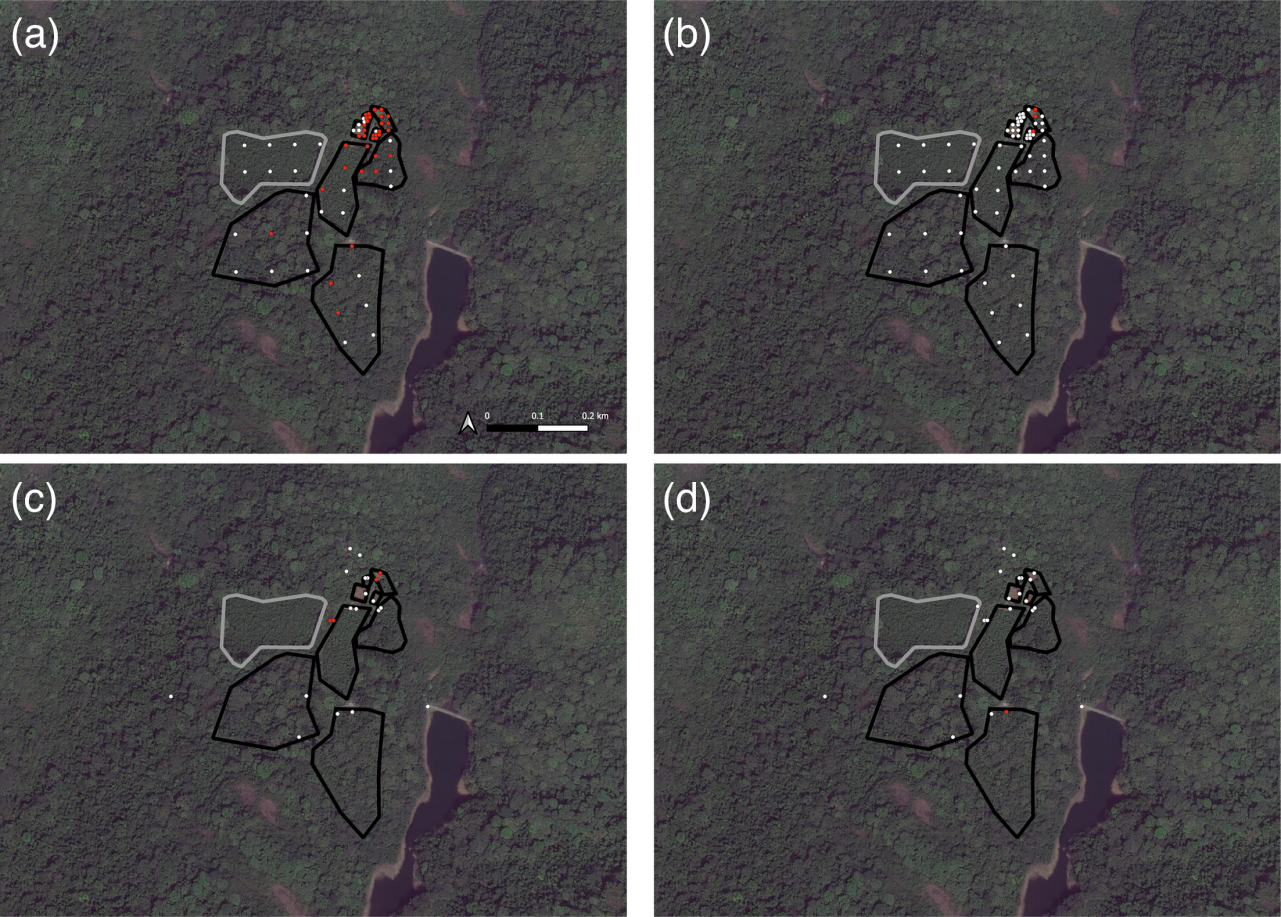
Map of chimpanzee enclosures at TCS in Western Area National Park, Sierra Leone. Polygons (black and grey) indicate chimpanzee enclosure perimeters (*n*=9) inside of which soil samples were collected. Chimpanzees have been absent from one enclosure (grey polygon) for several years. Dots indicate PCR-positive samples for *S. troglodytae* (red) or negative samples (white). (a) Soil (*n*=63) tested in the dry season. (b) Soil (*n*=63) tested in the rainy season. (c) Water (*n*=21) tested in the dry season. (d) Water (*n*=23) tested in the rainy season. Figure created using QGIS v. 3.28.7 with basemap from ESRI World Imagery.

*S. troglodytae* environmental prevalence during the dry season ranged from 4.8% (95% CI: <0.01, 25.41) in food items to 54% (95% CI: 41.79, 65.69) in soil. During the rainy season, prevalence ranged from 0% (95% CI: 0, 18.98) in food items to 8.7% (95% CI: 1.25, 27.97) in water samples. We detected *S. troglodytae* in soil from each of the eight inhabited enclosures (four non-forested and four forested) during the dry season but in only two of these same enclosures, both non-forested, during the rainy season (Fig. 3). We did not detect *S. troglodytae* in a forested enclosure where chimpanzees had not been housed for several years (i.e. since 2017) during either season. In water samples, we detected *S. troglodytae* more frequently during the dry season (19%, 95% CI: 7.08, 40.59) than the rainy season (8.7%, 95% CI: 1.25, 27.97) (Fig. 3). Positive water samples included taps or ponds within enclosures (both seasons), as well as water storage tanks (dry season only). The only food sample that tested positive for *S. troglodytae* was an unwashed fruit in the dry season.

Results of a binomial GLM indicated that chimpanzee faeces were 62.8 times (95% CI: 11.31, 1202.67) more likely to test positive for *S. troglodytae* during the dry season compared to the rainy season (Table 2). Additionally, individuals in forested enclosures were 5.48 (95% CI: 1.45, 23.58) times more likely to test positive for *S. troglodytae* than those in non-forested enclosures and 26.63 (95% CI: 1.75, 830.49) times more likely than those in quarantine. Neither age nor sex had a significant effect on the probability of *S. troglodytae* detection in chimpanzees at TCS. A second binomial GLM showed that environmental samples were 14.35 (95% CI: 5.68, 44.32) times more likely to test positive for *S. troglodytae* in the dry season than in the rainy season (Table 2). The odds of detecting *S. troglodytae* in soil samples at TCS was 3.06 (95% CI: 1.16, 9.19) and 21.96 (95% CI: 4.26, 403.74) times higher than in water or food samples, respectively.

**Table 2.** Results of (a) binomial GLM for effects of season, age, sex and enclosure type on *S. troglodytae* presence/absence in TCS chimpanzees and (b) binomial GLM for effects of season and sample type on *S. troglodytae* presence/absence in TCS environment

(a) Predictor	*β*	se	Wald *X*^2^ test	*P*
(Intercept)	0.28267	0.68282	–	–
Dry season†	4.14002	1.08430	3.818	<0.001***
Age	0.01995	0.03549	0.562	0.574
Sex‡	0.90482	0.60987	1.484	0.138
Non-forested enclosure§	−1.70196	0.70071	−2.429	0.015*
Quarantine enclosure§	−3.28219	1.48800	−2.206	0.027*
**(b) Predictor**	***β***	**se**	**Wald *X*^2^ test**	***P***
(Intercept)	−2.5931	0.4664	–	–
Dry season†	2.6640	0.5147	5.176	<0.001***
Water¶	−1.1190	0.5209	−2.148	0.032*
Food¶	−3.0892	1.0492	−2.944	0.003**

*P<0.05; **P<0.01; ***P< 0.001.

†Reference category for season: rainy.

‡Reference category for sex: female.

§Reference category for enclosure type: forested.

¶Reference category for sample type: soil.

Results of genus-wide, pan-*Sarcina* PCR testing (i.e. testing for all members of the genus *Sarcina*, not only *S. troglodytae*) showed that this genus of bacteria was ubiquitous in chimpanzee faecal samples from TCS during the dry season (96.7%, 95% CI: 87.97, 99.75) and the rainy season (93.1%, 95% CI: 83.09, 97.76) (Fig. S2). We detected bacteria of the genus *Sarcina* in soil samples from eight of the nine enclosures in both seasons at a similar prevalence, 57.1% (95% CI: 44.85, 68.61) in the dry season and 52.4% (95% CI: 40.27, 64.22) in the rainy season (Fig. S3). Interestingly, we did not detect bacteria of the genus *Sarcina* in the forested enclosure from which chimpanzees had been absent for several years, as we found for *S. troglodytae*. We detected bacteria of the genus *Sarcina* in water samples from inside and outside of chimpanzee enclosures at TCS less frequently in the dry season (23.8%, 95% CI: 10.23, 45.49) than in the rainy season (43.5%, 95% CI: 25.61, 63.21) (Fig. S3). The prevalence of bacteria of the genus *Sarcina* was lower in food samples during the dry season (15%, 95% CI: 4.39, 36.88) than during the rainy season (25%, 95% CI: 10.81, 47.25).

### TCRC

*S. troglodytae* was not detected anywhere at TCRC (overall prevalence: 0%, 95% CI: 0, 2.24) despite sample sizes comparable to those tested at TCS: chimpanzee faeces (*n*=77), monkey faeces (*n*=17), soil (*n*=82), water (*n*=9) and food (*n*=18). In contrast, bacteria of the genus *Sarcina* were detected in all sample types except for food. Nearly all (97.5%, 95% CI: 90.69, 99.84) chimpanzee faecal samples, including individuals from all three islands and all groups at the main sanctuary, were positive for bacteria of the genus *Sarcina* (Fig. S2). We detected bacteria of the genus *Sarcina* in soil samples from all four enclosures and all three islands at TCRC at a prevalence (53.7%, 95% CI: 2.92, 64.05) similar to that at TCS (Figs. S2 and S4). Two of nine (22.2%, 95% CI: 5.34, 55.72) water samples, an island water source in the forest and a pond in a non-forested enclosure at the main sanctuary, were positive for bacteria of the genus *Sarcina*. Additionally, we detected bacteria of the genus *Sarcina* in 100% (95% CI: 78.37, 100) of monkey faecal samples.

### NICS, KNP and LMNP

We detected *S. troglodytae* in chimpanzee faecal samples from NICS (10.9%, 95% CI: 4.29, 23.49) and KNP (2.6%, 95% CI: <0.01, 14.7) but at far lower prevalences than TCS. There was no association between sex and *S. troglodytae* detection at NICS or KNP (Table S2). We did not detect *S. troglodytae* in chimpanzee faeces (*n*=18) or soil samples (*n*=19) from LMNP (overall prevalence 0%, 95% CI: 0, 7.43). In contrast, we detected bacteria of the genus *Sarcina* in chimpanzee faecal samples from NICS (100%, 95% CI: 90.8, 100), KNP (68.4%, 95% CI: 52.45, 81.01) and LMNP (66.7%, 95% CI: 43.57, 83.9) and in soil samples (21.1%, 95% CI: 7.95, 43.89) from LMNP (Fig. S2).

### Combined analyses

A binomial GLM assessing the effects of study site, age and sex on *S. troglodytae* detection showed that chimpanzee faecal samples from TCS were significantly more likely to test positive for *S. troglodytae* than those from NICS (odds ratio: 44.42; 95% CI: 14.98, 161.73) or KNP (odds ratio: 366.14; 95% CI: 48.3, 8912.43) (Table 3). Additionally, for each 1-year increase in chimpanzee age, the model predicts a 5% increase in the odds of *S. troglodytae* detection (odds ratio: 1.05; 95% CI: 1.01, 1.1).

**Table 3.** Results of a binomial GLM for effects of study site, age and sex on *S. troglodytae* presence/absence in TCS, NICS and KNP chimpanzees.

Predictor	*β*	se	Wald *X*^2^ test	*P*
(Intercept)	0.50764	0.38482	–	–
NICS†	−3.79377	0.60011	−6.322	<0.001***
KNP†	−5.90303	1.25510	−4.703	<0.001***
Age	0.05013	0.02334	2.147	0.032*
Sex‡	0.14948	0.39399	0.379	0.704

*P<0.05; **P<0.01; ***P< 0.001.

†Reference category for study site: TCS.

‡Reference category for sex: female.

A binomial GLM assessing the effects of study site on *Sarcina* detection in chimpanzees showed that faecal samples from TCS were significantly more likely to test positive for bacteria of the genus *Sarcina* than those from both wild chimpanzee populations, KNP (odds ratio: 8.62; 95% CI: 3.06, 26.79) and LMNP (odds ratio: 9.33; 95% CI: 2.56, 34.61) (Table S4). Results from a binomial GLM assessing the effects of sample type and study site on *Sarcina* detection in the environment at TCS, TCRC and LMNP indicated that the odds of detecting *Sarcina* in soil samples were larger than in sampled water (odds ratio: 2.73; 95% CI: 1.44, 5.33) or food (odds ratio: 7.62; 95% CI: 3.6, 18.19) (Table S4). Additionally, *Sarcina* was 5.07 (95% CI: 1.73, 18.49) times more likely to be detected in the environment at TCS compared to LMNP.

### DNA sequencing to confirm *S. troglodytae*

Sequences of 12 capsular biosynthesis PCR amplicons selected to represent all study sites and sample types confirmed * S. troglodytae*. Sequences were between 99.59% and 100% similar at the nucleotide level to an isolate from our original study cultured from the brain of a 13-year-old male chimpanzee at TCS who succumbed to ENGS in 2015 (GenBank acc. no. CP051754; [44]) and 98.75%–100% similar at the amino acid sequence level to this same isolate. A synonymous SNP at position 146 of the amplicon was observed in chimpanzee faecal samples from TCS and NICS (PQ092974 and PQ092975) resulting in an alanine to tyrosine amino acid substitution. All 12 sequences were deposited in GenBank (accession numbers PQ092974–PQ092985).

## Discussion

In a study of five chimpanzee populations in West, Central and East Africa, we found that *S. troglodytae* was 25.4 times more prevalent in sanctuary chimpanzees at TCS in Sierra Leone, the only site where ENGS has been recorded, than at all other sites combined (76.3% vs. 3%, respectively). At the other sites included in the study, *S. troglodytae* was either absent (TCRC and LMNP) or occurred at low prevalence in chimpanzees (10.9% at NICS and 2.6% at KNP). At TCS, * S. troglodytae* infection showed a seasonal pattern, with a higher proportion of chimpanzees testing positive during the dry season (96.7%) than during the rainy season (55.2%). *S. troglodytae* infection at TCS was also spatially heterogeneous, with a higher proportion of chimpanzees using forested enclosures testing positive (85.5%) than chimpanzees not using forested enclosures (63.3%). We identified soil as a potential reservoir of *S. troglodytae* at TCS, with 29.3% of soil samples testing positive overall. Mirroring the pattern in chimpanzee faeces, *S. troglodytae* was more commonly detected in soil during the dry season (54%) than during the rainy season (4.8%). These results for *S. troglodytae* were markedly different than for bacteria of the genus *Sarcina* in general. Broad-spectrum testing for bacteria of the genus *Sarcina* found members of this genus to be common in chimpanzees across Africa (overall prevalence of 87.8%) with a higher prevalence in sanctuary chimpanzees (93.1%–100%) than in wild chimpanzees (66.7%–68.4%).

Our findings indicate that *S. troglodytae* is ubiquitous in chimpanzees at TCS, even when clinical signs consistent with ENGS are not observed, and that this differentiates TCS from any of the other chimpanzee populations studied. We did not detect * S. troglodytae* in sanctuary chimpanzees at TCRC in Central Africa, where ENGS has not been reported. This finding is noteworthy because TCS and TCRC are outwardly similar sanctuaries in terms of their locations in humid, forested areas. We also did not find *S. troglodytae* in wild chimpanzees at LMNP in Sierra Leone, emphasizing that the bacterium’s distribution is not a simple function of geography. Reinforcing this observation, we found *S. troglodytae* at only low prevalence in chimpanzee populations in Uganda in East Africa, which are located in drier, cooler environments than those of TCS and TCRC. Overall, the markedly higher prevalence of *S. troglodytae* at TCS than anywhere else, including ecologically similar locations and geographically nearby locations, strengthens the epidemiological association between *S. troglodytae* and ENGS.

Notably, we observed a higher prevalence of *S. troglodytae* in faecal samples from chimpanzees at TCS collected during the dry season (December–April) compared to the rainy season (May–November). This finding concords with the peak seasonality of ENGS cases in March [44], further strengthening the association between *S. troglodytae* and ENGS. Seasonality in the prevalence of *Sarcina* is not unprecedented. A study of semi-free-living rhesus macaques in Guangxi, China, revealed seasonal differences in the gut microbiota such as the enrichment of three genera, including *Sarcina*, during the dry season, and this trend was attributed to a higher fibre diet during the dry season [58]. Dietary differences could also explain our finding of a higher *S. troglodytae* detection in individuals utilizing forested enclosures at TCS, who, unlike those utilizing non-forested enclosures or those in quarantine, routinely forage on fibre-rich natural vegetation in addition to consuming their normal diet of fruits, root vegetables, bulgur and boiled eggs provided by the sanctuary. More broadly, the gut microbiomes of wild gorillas and chimpanzees in the Republic of Congo have been shown to fluctuate seasonally in response to rainfall and diet [59]. Enteric bacterial pathogen prevalence in diarrhoeic children has been shown to vary seasonally in West and East Africa; however, seasonality may vary geographically [60,63]. In most cases, the mechanisms underlying these seasonal patterns are multifactorial, complex and poorly understood [64].

Of the environmental samples tested in this study, *S. troglodytae* was detected most frequently in soil collected during the dry season at TCS. There is evidence for soil as an environmental reservoir of many bacterial pathogens, including phylogenetically related clostridial pathogens (genus *Clostridium*) [44], such as *Clostridium perfringens* [50]. Other reservoirs of pathogenic clostridia include faeces, wastewater and food [65]. A critical component of disease transmission by clostridial pathogens is the formation of spores under environmental conditions which are unfavourable for vegetative cells [50]. Clostridial myonecrosis caused by *C. perfringens* occurs when spores from soil enter muscle tissue via a wound. Then, in the presence of germinants, these dormant spores germinate into toxin-releasing vegetative cells responsible for disease. *C. perfringens* has been shown to be more prevalent in faeces of lambs and calves in Egypt during the winter season, possibly due to poor hygienic conditions, lower temperature, higher humidity or pasture change [66,67]. Canine tetanus caused by *Clostridium tetani* was most commonly observed during the winter season in England, suggesting that these weather conditions may be favourable for spore formation or result in increased exposure to spore-containing soil due to wet and muddy conditions [68]. A concurrent study on canine tetanus in California did not reveal evidence of seasonality, perhaps due to greater climate variability in the study’s geographic region [69]. In contrast to our findings, clostridial diseases such as blackleg (*Clostridium chauvoei*) in cattle [70] and enterotoxemia (*C. perfringens*) in rhinoceroses [71,72] have been associated with increased rainfall, which may facilitate the spread of spores and vegetative cell growth and lead to abrupt changes in vegetation that alter the gut microbiota and allow for pathogen proliferation. While prior whole-genome sequencing of *S. troglodytae* did not demonstrate the presence of toxin genes [44], the identification of the bacterium in the environment at TCS and its close phylogenetic relationship to spore-forming clostridia suggest that sporulation may influence environmental persistence, prevalence and seasonality of *S. troglodytae*. Additionally, since we did not detect *S. troglodytae* in soil from a forested enclosure at TCS where chimpanzees had not been housed for several years, it is likely that the chimpanzee gastrointestinal tract serves as the primary reservoir for the bacterium and contributes to its presence and persistence in soil by frequent reintroduction from defecation.

In contrast to our findings for *S. troglodytae*, broad-spectrum testing for all members of the genus *Sarcina* revealed positive samples in all chimpanzee populations included in the study. Prior studies indicate that the genus appears to be often found in the gastrointestinal tract of clinically normal NHPs both in captive and wild settings. *Sarcina* has been detected in faecal samples from a clinically normal, wild mountain gorilla (*Gorilla beringei beringei*) in Uganda [73], semi-provisioned and laboratory-housed rhesus macaques in China [58,74] and numerous zoo-housed New and Old World monkeys in the Czech Republic and Slovakia [42,75, 76]. Additionally, *S. ventriculi* genomes were sequenced from isolates from the faeces of two clinically normal, wild Yakushima macaques (*Macaca fuscata yakui*) in Japan [77].

Dietary differences among chimpanzees at the sample sites included in our study may contribute to the reported differences in prevalence of carbohydrate-fermenting sarcinae [78,79]. Unlike *S. troglodytae*, the prevalence of *Sarcina* at the genus level did not differ seasonally at TCS, but we observed higher prevalence in sanctuary chimpanzee populations (TCS, TCRC and NICS) than in wild ones (LMNP and KNP), similar to findings comparing provisioned and wild rhesus macaque gut microbiota in China [80]. Likewise, the increased prevalence of *C. perfringens* has been documented in captive chimpanzees compared to wild counterparts and attributed to dietary and environmental differences [81]. In clinically normal humans, *S. ventriculi* was isolated from 75 out of 106 (70.8%) of faecal samples from individuals following a vegetarian diet compared to only 2 out of 123 (1.6%) of faecal samples from omnivores [41]. In a study on the gut microbiota of zoo-housed red pandas (*Ailurus fulgens*) across weaning stages, *Sarcina* was only present at the adult stage, at which point the diet had been fully transitioned from milk replacer to bamboo and leaf-eater biscuits [82].

Across sites, we also found that *Sarcina* spp. were commonly present in the environment. This was especially the case for soil samples from both TCS (54.8% overall) and TCRC (53.7%) and less so in water and food samples. The genus *Sarcina* has been isolated from soil, mud and sediment [83,85]; however, few studies have provided systematic data on its environmental presence. Our results provide further evidence for the ubiquitous nature of *Sarcina* in hosts and their environments. Soil plays a critical role in the persistence of clostridial pathogens where they exist in spore form, capable of surviving extreme or unfavourable conditions for potentially decades [86]. These bacteria, such as *Clostridium botulinum*, can be introduced into soil or other environmental reservoirs (e.g. wastewater) as a result of shedding from the gastrointestinal tracts of humans and other animal hosts [87]. While the sporulation ability of *S. troglodytae* is unclear, there is evidence of spore formation *in vitro* under alkaline conditions for other members of the genus *Sarcina* [88].

Broadly, these findings expand our knowledge of the role of sarcinae in human and animal health. Since the first observation of the genus *Sarcina* in 1842 [89], these bacteria have been widely detected in faecal samples from clinically normal humans [40,41], domestic animals [90,93] and wildlife [42,94]. Conversely, case reports in humans, which have increased dramatically in the last 15 years [44,95], indicate that *Sarcina*-associated disease (e.g. gastritis and gastric ulcers), often attributed to *S. ventriculi*, is frequently observed in individuals with comorbidities that result in delayed gastric emptying (e.g. pyloric stenosis and diabetes mellitus), which may allow the bacteria to thrive in an acidic environment [45,95,97]. *Sarcina*-associated infections have also been associated with sudden death and gastrointestinal disease (e.g. acute gastric dilation or abomasal bloat and gastrointestinal emphysema) in dogs [98], horses [98,99], young ruminants [100,106] and NHPs [44,46, 47]. Gas production by sarcinae as a result of carbohydrate fermentation appears to be clinically relevant, particularly in fatal cases of emphysematous gastritis or abomasitis [43,44, 107]. Additionally, data from human cases [108,110] and SIV-infected wild chimpanzees in Tanzania [48] suggest that *Sarcina* may thrive in immunocompromised individuals.

The results presented here, in combination with our original study [44], are similar to those reported by Lee *et al*. [46], who found that two laboratory rhesus macaques that died suddenly had *Sarcina* in non-alimentary tissues and severe gastric dilation on post-mortem examination; one also had emphysematous gastritis. Rectal swabs from 24 out of 26 (92.3%) unaffected macaques housed in the same facility tested positive for the genus *Sarcina* by PCR for the 16S rRNA gene. Likewise, *S. troglodytae* appears to be a typical member of the gut microbiota of clinically normal TCS chimpanzees but has also been associated with a disease syndrome, ENGS, in this population in which *S. troglodytae* has been detected in non-alimentary tissues of ENGS cases only [44]. Given the absence of *S. troglodytae* in chimpanzees and the environment at LMNP and TCRC, the bacterium’s geographic distribution appears to be much smaller than what we observed at the genus level, similar to what has been reported in humans and other NHPs worldwide. We speculate that *S. troglodytae* may be an opportunistic pathogen that is well adapted to environmental or host conditions at TCS, unlike other sites included in our study, given its high prevalence and identification in chimpanzees at this site since as early as 2013.

Members of the genus *Sarcina*, including the novel candidate species examined here, *S. troglodytae*, have been differentiated based on whole-genome sequencing and cell morphology [44,96]. Molecular diagnostic methods commonly used to identify members of the genus *Sarcina* in clinical samples are primarily limited to assays that target the 16S rRNA gene [45]. This approach may be unable to differentiate among *Sarcina* spp. as they share >99% 16S nucleotide sequence identity, a challenge that has been reported for the family *Clostridiaceae* [111,112]. Likewise, presumptive species identification based on morphology does not predict the pathogenicity of sarcinae, which may differ at the species and strain level [42,44, 95]. While *S. maxima* has not been associated with disease, to our knowledge, *S. ventriculi* is often considered an opportunistic gastrointestinal pathogen [75].

We speculate that the genus *Sarcina* may contain diverse, uncharacterized taxa that vary along a spectrum of pathogenicity, from benign commensals to opportunistic or even frank pathogens. We recommend that future investigations of *Sarcina*-associated disease expand beyond describing the characteristic morphology of the genus and conduct additional analyses (e.g. molecular identification and biochemical assays) to identify the particular species or strains of the bacterium associated with disease. Only with a critical mass of such efforts will it become clear which sarcinae merit concern as agents of human and animal disease, and only then can specific diagnostic assays be designed. In this light, it remains intriguing that ENGS cases have not been reported in people working at TCS, even though many animal care staff come into close contact with TCS chimpanzees, their waste products and their environment. With future investigations as described above, it should be possible to make inferences about host specificity of the various sarcinae known and yet to be discovered.

We acknowledge certain inherent limitations of our findings. For example, although we did not detect *S. troglodytae* in samples from LMNP and TCRC, it is possible that the bacterium is present at these locations at very low prevalence or below the lower limit of detection of our assay (26–30 gene copies per reaction; Table S5). Similarly, we note that the primers for our species-specific PCR target a *S. troglodytae* capsular biosynthesis gene, but the extent of natural variation in this gene is unknown such that sites where we failed to detect *S. troglodytae* could still harbour the bacterium if this gene is absent [113,114]. Additionally, samples from LMNP and TCRC could only be collected during the rainy season; thus, we cannot comment on seasonal variation at these sites, and *S. troglodytae* prevalence was higher during the dry season at TCS. Finally, our methods are unable to determine whether *S. troglodytae* detected in faeces is actively replicating in the gastrointestinal tracts of positive animals, which would be a prerequisite for linking the bacterium to ENGS pathology [44]. However, evidence from many other studies that sarcinae were present and growing at the sites of histopathologic lesions [46,47, 98, 103, 105] supports the idea that *S. troglodytae* is not merely ‘passing through’ the chimpanzee gastrointestinal tract.

Sierra Leone has been identified as an important site for conserving the critically endangered western chimpanzee [39,115, 116]. As part of these efforts, TCS has worked to rescue and rehabilitate juvenile chimpanzees in Sierra Leone from the illegal bushmeat and pet trades since 1995. Over time, the sanctuary population has grown to almost 120 individuals, and this has required additional enclosures to be built to maintain welfare as facility capacity is reached [39,116]. Sanctuaries in Guinea and the Republic of Congo have reintroduced sanctuary chimpanzees to protected, wild areas [117,120], although the practice is controversial and challenging. Additionally, TCRC has successfully relocated three groups of chimpanzees to forested islands where they remain cared for and provisioned by sanctuary staff. Given the lack of evidence thus far for *S. troglodytae* in wild chimpanzees at LMNP, a critical site for western chimpanzee conservation [51], and knowledge of wild chimpanzees living in Western Area Peninsula National Park surrounding TCS, we caution against relocation efforts that could potentially introduce an opportunistic pathogen to naïve, wild chimpanzee populations or other species [121]. Such concerns might be modified (in either direction) by future studies of the mechanistic relationship (if any) between *S. troglodytae* and ENGS.

In light of growing evidence for pathogenic *Sarcina* infections in humans [45] and captive NHPs [46,47], our findings build upon previous findings [44] to provide further support for an epidemiologic association between *S. troglodytae* and ENGS. They have implications for the management of sanctuary chimpanzees, which is part of broader chimpanzee conservation efforts. Additionally, our methods could be used for surveillance of *S. troglodytae* over time or in additional chimpanzee populations and for investigating future suspected ENGS cases and cases of similar disease. Future studies to elucidate the role of *S. troglodytae* in the aetiology of ENGS and to develop effective preventions and treatments will likely require experimental studies in laboratory animals (e.g. rodents), if model animal systems can be developed.

## Supplementary material

10.1099/jmm.0.002044 Uncited Supplementary Material 1.

10.1099/jmm.0.002044 Uncited Supplementary Material 2.

## Abbreviations

ENGS: epizootic neurologic and gastroenteric syndrome
GLMs: generalized linear models
KNP: Kibale National Park
LMNP: Loma Mountains National Park
NHP: non-human primate
NICS: Ngamba Island Chimpanzee Sanctuary
SIV: simian immunodeficiency virus
TCRC: Tchimpounga Chimpanzee Rehabilitation Center
TCS: Tacugama Chimpanzee Sanctuary
