# Supplementary material for: Distribution and prevalence of Sarcina troglodytae in chimpanzees and the environment throughout Africa
Source: J Med Microbiol. 2025 Jul 18;74(7):002044. doi: 10.1099/jmm.0.002044 (PMC12284408; doi:10.1099/jmm.0.002044)

Supplementary Figure 1: Gel image of (A) *S. troglodytae* and (B) pan-*Sarcina* PCRs

1: Feces from an affected chimpanzee at TCS (case from Owens et al., 2021)

2: *S. troglodytae* liquid culture from brain of ENGS affected chimpanzee at TCS (case from Owens et al., 2021)

3: *Sarcina ventriculi* colony ATCC 29068

4: *Sarcina maxima* liquid culture ATCC 33910

N: No template

Ladder: 1kb Plus DNA Ladder (New England Biolabs)

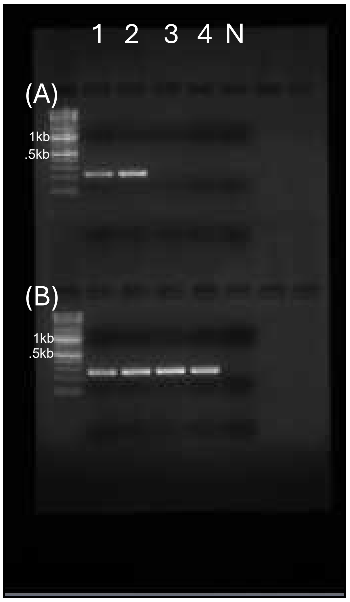

Supplementary Figure 2. Prevalence of bacteria of the genus *Sarcina* in (A) chimpanzee feces at TCS, TCRC, NICS, KNP, and LMNP and (B) the environment at TCS, KNP, and LMNP.

A)

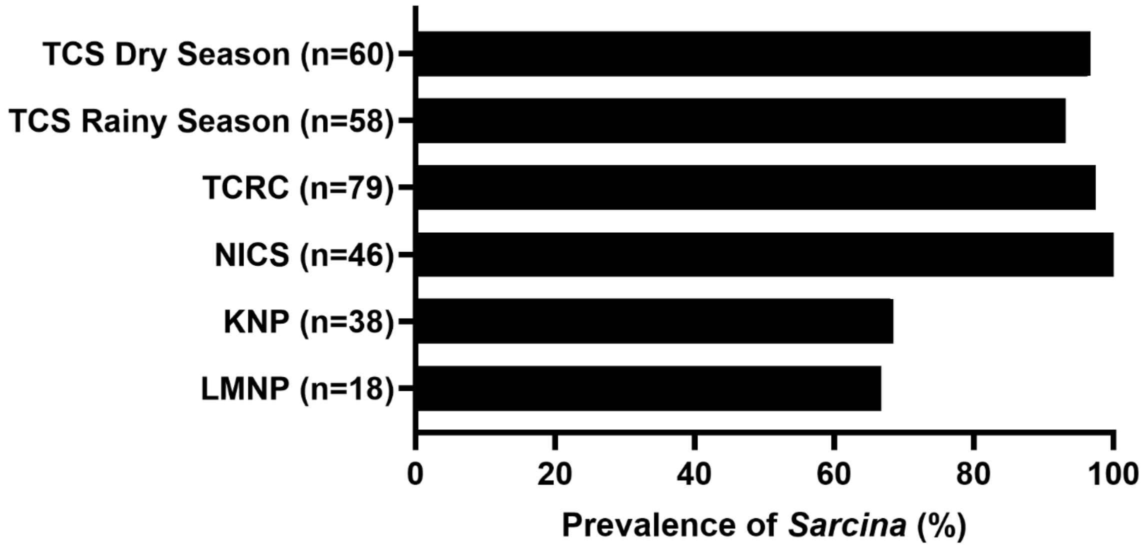

B)

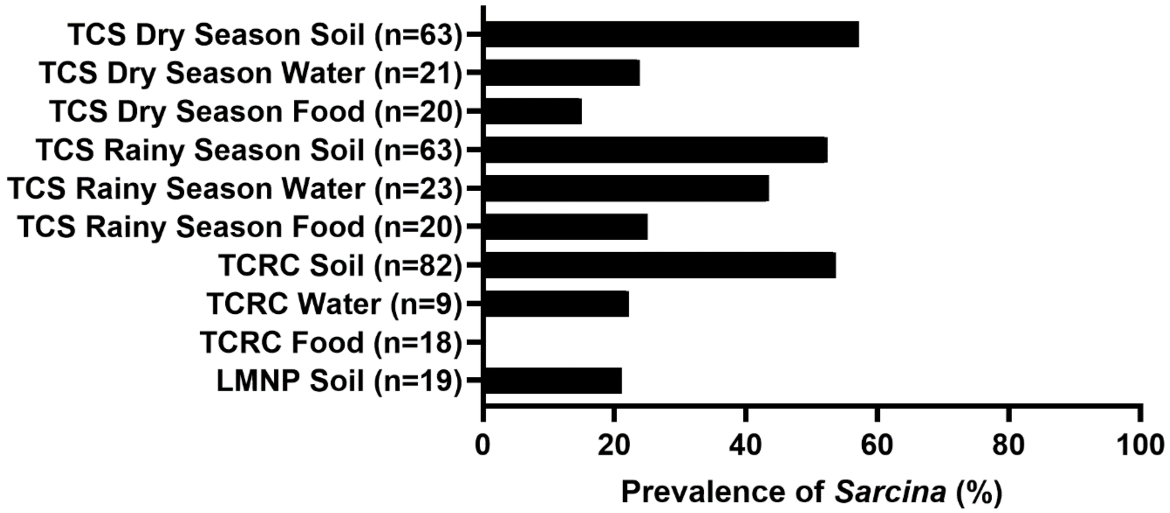

Supplementary Figure 3. Map of chimpanzee enclosures at Tacugama Chimpanzee Sanctuary within Western Area National Park, Sierra Leone. Polygons (black and gray) indicate chimpanzee enclosure perimeters (n=9) inside of which soil samples were collected. Chimpanzees have been absent from one enclosure (gray polygon) for several years. Dots indicate PCR-positive samples for any bacteria of the genus *Sarcina* (red) or negative samples (white). A: soil (n=63) tested in the dry season. B: soil (n=63) tested in the rainy season. C: water (n=21) tested in the dry season. D: water (n=23) tested in the rainy season. Figure created using QGIS v. 3.28.7 with basemap from ESRI World Imagery.

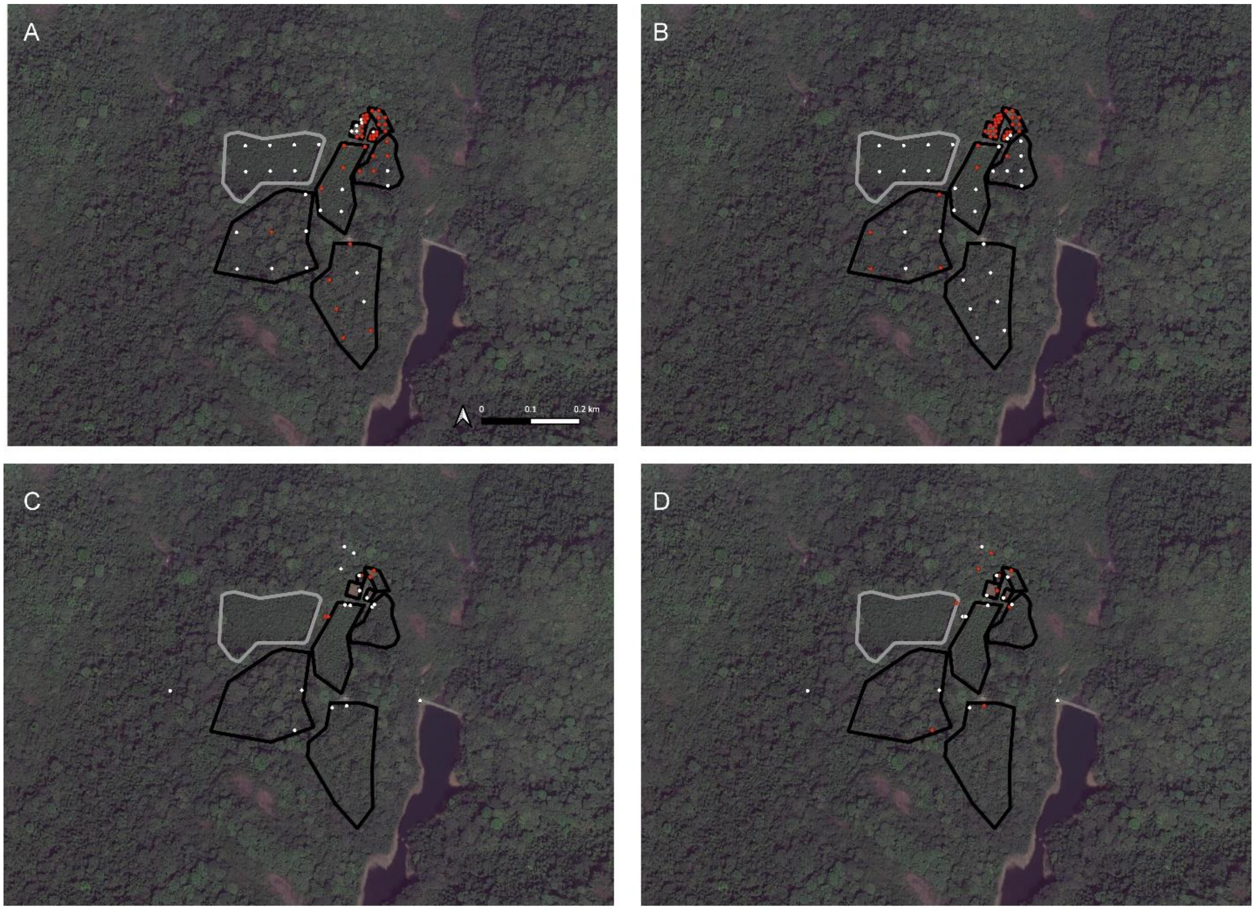

Supplementary Figure 4. Maps of A: chimpanzee enclosures at Tchimpounga Chimpanzee Rehabilitation Center in Tchimpounga Nature Reserve, Republic of Congo. B: Ngombe, Tchindzoulou, and Tchibebe islands, part of Tchimpounga Chimpanzee Rehabilitation Center, in the Kouilou River, Republic of Congo. Polygons (black) indicate enclosure perimeters inside of which soil samples were collected. Dots indicate PCR-positive samples for any bacteria of the genus *Sarcina* (red), negative samples (white), and points not tested due to inaccessibility (gray). A: soil (n=28) tested at the main sanctuary. B: soil (n=54) tested at the islands. Figure created using QGIS v. 3.28.7 with basemap from ESRI World Imagery.

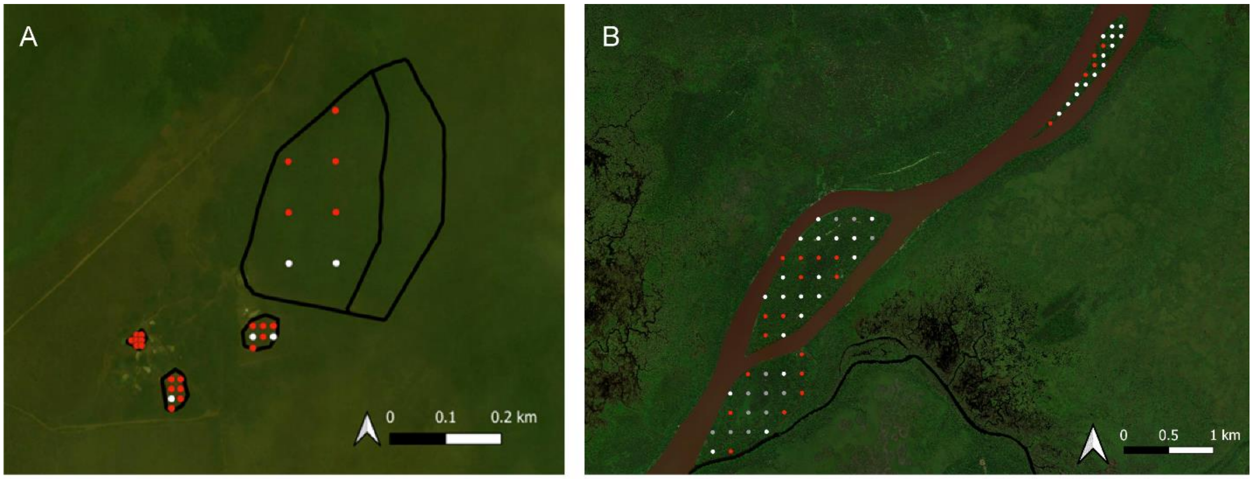

Supplement: Uncited Supplementary Material 1. [file jmm-74-02044-s001.pdf]
